# Investing in health R&D: where we are, what limits us, and how to make progress in Africa

**DOI:** 10.1136/bmjgh-2018-001047

**Published:** 2019-03-04

**Authors:** Victoria Simpkin, Evelyn Namubiru-Mwaura, Lorcan Clarke, Elias Mossialos

**Affiliations:** 1 Department of Health Policy, London School of Economics and Political Science, London, UK; 2 Africa Centre, Stockholm Environment Institute, Nairobi, Kenya; 3 Institute of Global Health Innovation, Imperial College London, London, UK

**Keywords:** Investment, research, research capacity, health, Africa

## Abstract

Global research and development (R&D) pipelines for diseases that disproportionately affect African countries appear to be inadequate, with governments struggling to prioritise investment in R&D. This article provides insights into the sources of investment in health science research, available research capacity and level of research output in Africa. The African region comprises 15% of the world’s population, yet only accounted for 1.1% of global investments in R&D in 2016. There were substantial disparities within the continent, with Egypt, Nigeria and South Africa contributing 65.7% of the total R&D spending. In most countries of the Organisation for Economic Co-operation and Development, the largest source of R&D funding is the private sector. R&D in Africa is mainly funded by the public sector, with significant proportions of financing in many countries coming from international funding. Challenges that limit private sector investment include unstable political environments, poor governance and corruption. Evidence suggests various research output and research capacity limitations in Africa when considering a global context. Metrics that reflect this include university rankings, number of researchers, number of publications, clinical trials networks and pharmaceutical manufacturing capacity. Within the continent there are substantial regional disparities. Incentivising investment is crucial to foster current and future research output and research capacity. This paper outlines some of the many commendable initiatives under way. Innovative and collaborative financing mechanisms can stimulate further investment. Given the vast inequalities across Africa in R&D, strategies need to reflect the different capacities of countries to address this disparity.

**Summary box**

- Health science research, funding and research capacity are insufficient to address Africa’s current unmet health needs, and there are ambitious country-set targets and frameworks for progress still to be met over the coming decade.
- There are substantial disparities in within-continent research and development (R&D) investment; estimates from 2016 indicated South Africa, Egypt and Nigeria contributed almost two-thirds of the total domestic spending on R&D in Africa.
- Measures of R&D outputs and capacity suggest both inter-regional and intraregional disparity based on investments, university rankings, number of researchers, number of publications, patent holdings, clinical trial networks and pharmaceutical manufacturing capacity.
- Disparities in R&D capacity within Africa suggest the likely value of support for collaborative science, technology and innovation networks between African nations, with any new partnerships harnessing the substantial momentum of R&D initiatives that already exist.
- Development of clear and context-relevant financing strategies and mechanisms can foster further public, private and international investment in R&D across the region.

## Introduction

Africa produces about 2% of world research output, yet the region, as defined by the Unesco, accounts for 15% of the global population and 25% of the global disease burden.^1^ Research and development (R&D) pipelines for diseases that disproportionately affect African countries and address Africa’s unmet health needs are insufficient.^2^ More needs to be done. This includes leveraging investment and supporting capacities for health science research across Africa.

Many African countries have adopted targets that reflect aims of improved prosperity and achieving middle-income country status in the coming decade. Science, technology and innovation are key to these goals.^3^ As themes, they are central pillars to the African Union’s Science Technology and Innovation Strategy for Africa (STISA 2024) and Agenda 2063.^4–6^ Infrastructure, financial and knowledge resources in African nations present a diverse set of challenges to investment in R&D.^7^ Yet rising gross domestic product (GDP) coupled with young, and growing, populations and increasing urbanisation in many countries may continue to drive growth in Africa and capacity for R&D growth.^8^ Health science research can play a central role in this.

This analysis offers an overview of funding and capacities for health science research and research capacity in Africa. It highlights challenges, opportunities and recommendations for progress. Our analysis is informed by a semi-systematic literature review and expert interviews.

The literature review examined peer-reviewed and grey literature obtained using relevant search terms in online databases, with additional resources obtained through citations and institutional publication archives. Only English-language literature was reviewed, leaving scope for further review of evidence from French-speaking or Arabic-speaking authors. We undertook semi-structured interviews with nine representatives from governmental, non-governmental and academic institutions to enrich the scope of our research and engage further relevant literature (online supplementary appendix A for further information).^9^ Interviewees were selected using a judgement sampling approach.^10^

10.1136/bmjgh-2018-001047.supp3 Supplementary data

## Support for African health science research

### R&D funding across Africa

In 2016, Africa accounted for 1.1% (US$22.3 billion) of global investments in R&D.^11^ Egypt, Nigeria and South Africa accounted for 65.7%, or US$14.66 billion, of Africa’s total R&D spending.^11^

In 2007 African Union countries committed to investing at least 1% of GDP in R&D. This recognised the importance of R&D to sustainable development and the need to address Africa’s health needs. This goal has remained unrealised. Across sub-Saharan Africa the average share of GDP devoted to R&D activities was only 0.4% in 2015, or the most recent available year.^12^ Countries closer to the 1% target included Egypt, Kenya, Mali, Morocco, South Africa, and Tunisia while countries including Algeria, Cabo Verde and Lesotho invested less than 0.1% of GDP in R&D (online supplementary file A).^13^ R&D intensity is a sentinel indicator for economic policy. However, data on R&D investment are extremely limited for many African countries (see table 1).^13–15^

10.1136/bmjgh-2018-001047.supp1 Supplementary data

**Table 1:** Levels of gross domestic expenditure in R&D (% of GDP)

Expenditure on R&D (% of GDP)	Countries
>0.6	Egypt, Kenya, Malawi, Mali, Morocco, South Africa, Tunisia
0.4–0.6	Ethiopia, Gabon, Mozambique, Senegal, Tanzania, Uganda
0.2–0.4	Botswana, Ghana, Nigeria, Seychelles, Sudan, Togo, Zambia
0.1–0.2	Burkina Faso, Burundi, Gambia, Mauritius, Namibia
0<0.1	Algeria, Cabo Verde, Democratic Republic of the Congo, Lesotho, Madagascar
No data	Angola, Benin, Cameroon, Central African Republic, Chad, Comoros, Congo, Cote d’Ivoire, Djibouti, Equatorial Guinea, Eritrea, Guinea, Guinea-Bissau, Liberia, Libya, Mauritania, Niger, Rwanda, Sao Tome and Principe, Sierra Leone, Somalia, South Sudan, Swaziland, Zimbabwe

Sources: refs^13–15^.

GDP, gross domestic product; R&D, research and development.

### Sources of funding

The landscape of institutions funding African R&D is complex. Sources for domestic R&D include public sector, private sector and international funding. A country or region’s relative GDP devoted to R&D activities is known as gross expenditure on research and development (GERD). R&D intensity is conventionally measured as the ratio of GERD to GDP. Most of the world’s largest developed economies have overall levels of R&D expenditure exceeding 2% of GDP. In Africa, almost all countries invest less than 1%.^16^

**Figure 1:**
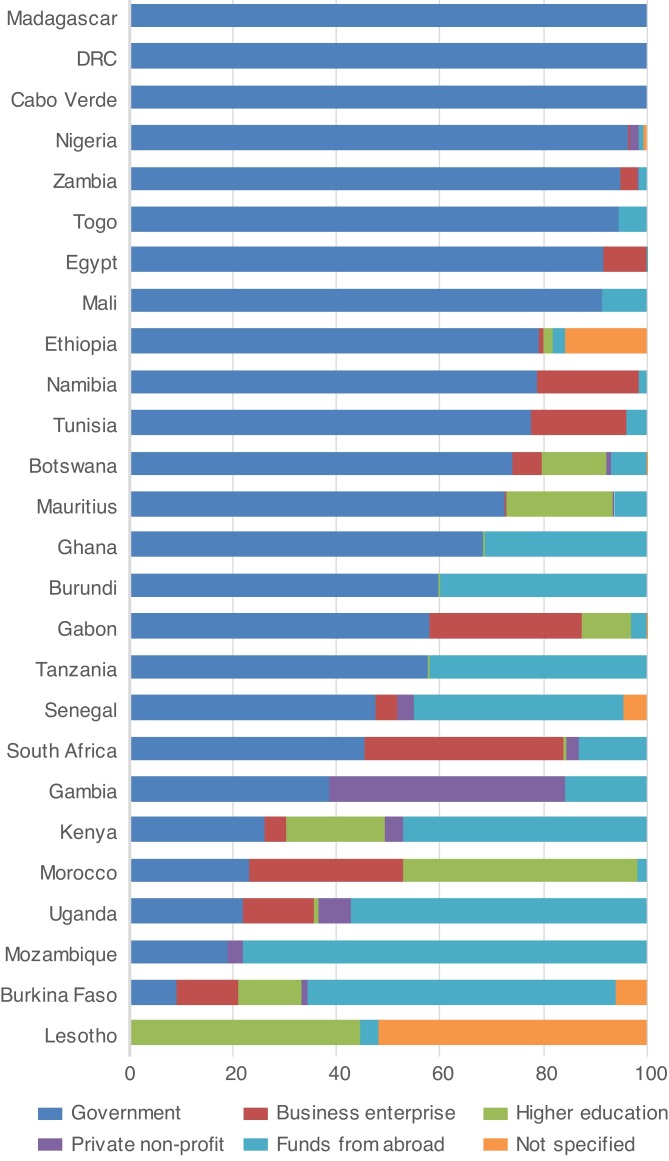
Source of funding for gross expenditure on research and development by country. Source^13^: (Data from 2014 or the latest available year).

Although GDP per capita and GERD per capita have been rising in most African countries, levels are low by world standards, and there is a marked disparity within the continent.^17^ Public investments in strengthening research capacity require extensive capital resources. However, rates of return are often unpredictable, and it is difficult to prioritise spending versus pressing government priorities in education, health and infrastructure. In most countries of the Organisation for Economic Co-operation and Development, the largest source of R&D funding is the private sector. African R&D has historically been mainly funded by the public sector, with international sources forming a substantial proportion of expenditures in many countries (see figure 1). For instance, foreign sources contributed significantly to 2015 R&D expenditures in Ghana (31%), Senegal (41%) and Burkina Faso (60%).^15^ South Africa has been an exception, hosting substantial private sector support.^17^ Encouraging governments to increase public funding of R&D and incentivising strong private sector engagement in the funding and performance of R&D activities remain a key regional challenge.

Globally, pharmaceutical companies are among the top investors in R&D in the health science sector, but this is not the case in Africa. Few African companies have R&D units or R&D directors to oversee product development and technology transfer.^3^

## Current capacities

According to Unesco, Africa had an estimated 198 researchers, in all fields, per million inhabitants in 2014.^13^ This compares with 428 in Chile and more than 4000 in the UK and the USA.^13^ Within Africa there is further disparity between countries. The top three countries with full-time researchers holding PhDs or equivalent per million people were all in North Africa: Tunisia, Morocco and Egypt. An estimated 878 researchers per million inhabitants in North Africa compares with 88 per million inhabitants in sub-Saharan Africa.^13^

Furthermore, many African researchers leave the continent. Over 10% of sub-Saharan Africans with graduate degrees emigrate. The numbers are even higher in the health workforce.^17^ Skilled researchers leave for several reasons. These can include a lack of local funding and training opportunities compared with high-income countries outside the region, which may have better research environments and working conditions. These reasons are common among regions experiencing a drain in research capacities.^18^

Retaining professionals with specific technical skills is essential to health research. However, other capacities are also essential to launch and sustain health research programmes. This involves training professionals to undertake administrative activities and hiring dedicated administrative staff. Key administrative activities for research include budget management, grant acquisition and procurement. Further dedicated support is essential for tasks involving human resource management, maintenance and legal support.^18^

There have been specific attempts to address emigration and harness the capacity of the African diaspora. The Africa Capacity Building Initiative, led by the Royal Society and UK Department for International Development (DFID), and the Carnegie African Diaspora Fellowship Program are two programmes guiding research expertise of African diaspora to the continent and helping to develop research and training capacities. The International Organization for Migration’s Migration for Development in Africa Diaspora Database and the Unesco-Hewlett Packard Brain Gain Initiative offer similar support.^19–22^ Further activities are outlined under the Initiatives and coalitions section of this paper.

### Training more health science researchers in Africa

In 2016 the average tertiary enrolment rate in Africa, the percentage of high school graduates enrolling in university, was 7.1%. The global average in 2016 was 25.1%.^23^ However, the growth trend is positive, with returns on investment in higher education in Africa estimated at 21%—the highest in the world.^3 24^ A 2015 report by the Africa-America Institute estimated that a 1-year increase in average tertiary education levels could raise annual GDP growth across Africa by 0.39%, potentially yielding an eventual increase in GDP of up to 12%.^25^

Sustainable health science research and research capacity building require strong scientific talent and high-quality universities. Only 26 universities in Africa featured in the Times Higher Education’s World University Rankings 2016–2017 (out of 980).^26^ Within the continent, the top 15 ranking universities span 7 countries (see table 2).^26^ One of the challenges that African universities face is the shortage of highly qualified researchers and other staff; a 2013 United Nations Economic Commission for Africa survey of nine African universities revealed that less than 50% of researchers and lecturers held PhDs.^3^

**Table 2:** Top 15 universities in Africa 2016–2017 (Times Higher Education Rankings)

Ranking	University	Country
1	University of Cape Town	South Africa
2	University of Witwatersrand	South Africa
3	Stellenbosch University	South Africa
4	Makerere University	Uganda
5	University of KwaZulu-Natal	South Africa
6	University of Pretoria	South Africa
7	University of Ghana	Ghana
8	University of Nairobi	Kenya
9	Suez Canal University	Egypt
10	Alexandria University	Egypt
11	Cairo University	Egypt
12	University of Marrakech Cadi Ayyad	Morocco
13	University of South Africa	South Africa
14	University of Ibadan	Nigeria
15	Mohammed V University of Rabat	Morocco

Source: ref^26^.

International initiatives have looked to address these issues. Examples include the World Bank establishment of the African Higher Education Centers of Excellence project, which aims to address regional development challenges and strengthen research capacity.^27^ Attracting foreign universities represents an opportunity for low-income and middle-income countries to engage in international technology transfer and bolster learning. For example, in 2011, a new campus of Carnegie Mellon University launched in Rwanda with funding from the Rwandan Government and the African Development Bank.^28^

### Collaboration across the region

In African countries researchers produce most publications with international coauthors, rather than with local coauthors. Most coauthors hail from institutions outside Africa. Nigeria and Egypt have been exceptions, with researchers producing most publications with domestic coauthors, 71% and 57%, respectively.^29^ See online supplementary file B for further details on this trend.

10.1136/bmjgh-2018-001047.supp2 Supplementary data

Africa’s regional research capacity has a fragmented appearance, with limited collaboration between subregions. Inter-regional collaboration (without any South African or international collaborator) comprises 2% of all East African research, 0.9% of West and Central Africa, and 2.9% of Southern Africa (online supplementary file B).^30^ A 2009 study of the Southern African Development Community (SADC) found only 5% of SADC papers published between 2005 and 2008 were coauthorships between an SADC researcher and another African researcher.^31^

Collaborative academic networks tend to be driven by funding availability, which may have biased research towards collaborations with researchers outside Africa. The main collaborating institutions are in the USA, the UK and France, the countries that are also the largest funders of research in biosciences in Africa. There is an increasing trend towards collaboration on health research within Africa, particularly for malaria research. However, this is still much lower than collaboration with institutions in Europe and the USA.^2 3^

Cross-sector collaboration can foster knowledge transfer and alternative funding channels. Table 3 presents coauthored publications as a proxy for cross-sector collaboration, as a relative percentage of each region’s total output between 2003 and 2012.^30^ Across each region, academic–corporate collaboration accounts for only a small percentage of each region’s total output. Collaboration between the academic and corporate sector is mainly within health sciences and through collaborations with global pharmaceutical companies.^30^

**Table 3:** Cross-sector collaboration as a percentage (%) of total publications for sub-Saharan African regions and comparator institutions, 2003–2012

Region	Academic–corporate (%)	Academic–government (%)	Academic–medical (%)
East Africa	2.4	17.2	6.0
Southern Africa	2.4	17.4	7.5
West/Central Africa	1.0	10.5	4.2
South Africa	2.8	12.6	3.0

Source: ref^30^.

Despite the potential value of international collaborative projects with high-income countries, there is justifiable concern. Priorities may not align. African institutions face challenges with leadership and ownership of research, which can create longer term issues with sustainability. In 2011, 38 African centres from across the continent were recognised as African Network for Drugs and Diagnostic Innovation (ANDI) Centres of Excellence in health innovation. This will establish an alliance of African institutions with the expertise and resources to progress health innovation and to encourage intra-African, South–South and North–South networking and collaboration.^32 33^

### Research outputs

Bibliometric indicators offer proxy measures, through the number of publications and citations, to assess the performance and influence of scientific research. Evidence suggests an estimated 60% growth in publications with African authors between 2008 and 2014.^3 17^ However these research outputs appear to be unevenly distributed at the country and subregional levels.^34 35^

Publications alone, as an indicator of research output, may give an incomplete picture. It omits a significant level of production of non-academic research output. Surveys by the Health Research System Analysis Initiative indicate that, despite low research production in sub-Saharan Africa, there is significant research activity, and institutions make an effort to disseminate their research to the intended audience.^36^ Other important dissemination dimensions include publication in regional and national journals and editing working papers.^36^ Interviewees noted that some African academics may publish research in local journals that are not indexed by the Institute for Scientific Information’s Web of Science. These journals may publish in a local language, as they target the local audience. Web of Science targets the international audience and is biased towards English-language content.

Countries in Europe and sub-Saharan Africa are partnering to increase research outputs through initiatives such as the European and Developing Countries Clinical Trials Partnership (EDCTP). The EDCTP aims to alleviate the health and economic burden of infectious diseases in Africa, with a focus on phase II and III clinical trials.^37^ The EDCTP focuses on multinational, multicentre projects. It has established regional networks for conducting clinical trials and promoting clinical research in sub-Saharan Africa. This promotes collaboration and sharing of expertise in resource-limited settings. Despite success stories, challenges remain. EDCTP projects receive limited contributions from African governments, with a heavy reliance on funding from Europe; this negatively impacts African ownership of the research. There has also been difficulty transferring research funds to and between institutions in some cases.^37^

### Pharmaceutical R&D

The commercial sector is key to health science R&D. Thirty-seven African countries have some pharmaceutical production, but few produce active pharmaceutical ingredients and intermediates.^38^ These inequalities are replicated in the imbalance in global R&D outputs according to health needs. From 1975 to 2004, only 1.3% of the 1556 new chemical entities registered were meant for use in tropical diseases and tuberculosis, despite these diseases accounting for 12% of the global disease burden.^39^ A mix of regulatory and financial barriers to commercialisation, weak intellectual property rights and a lack of basic infrastructure disincentivise private sector investment.

Additionally, there are limited domestic capabilities to undertake the experimental development or translational research phase. Despite growing capacity for the third phase of clinical trials, the production and manufacturing phase of the value chain remains weak.^3^ Local production relies on imported active ingredients—despite Africa’s raw material wealth. Interviewees noted that local manufacturing in Africa could foster affordability of essential drugs, increase local job opportunities and reduce dependency on foreign support.

## Opportunities for progress

Available evidence suggests persistent low investment in health science research in Africa, with substantial gaps in research output and capacity compared with high-income countries and some emerging economies. Several initiatives seek to create opportunities and address barriers towards progress.

Key challenges to catalysing investment include a lack of ownership of research agendas, poor capacity retention and inadequate knowledge.^40^ Institutional weaknesses also lead to difficulties in catalysing research. These include corruption, governance issues and political instability. A 2015 Transparency International survey found 22% of Africans who encountered a public service in the past 12 months say they paid a bribe.^41^ Assessing governance capacity also offers important structure to considering methods to increase public and private sector investment in health science R&D. The Ibrahim Index, an independent index for governance quality in Africa, notes most countries show signs of an improved overall governance score over the past decade. However, over half of the 40 index countries have shown signs of slackening and even reversals in their progress.^42^

Several challenges limit private sector investment. These include unstable political environments, poor governance, weak regulatory structures and corruption. Public policies may exacerbate market weaknesses, such as the imposition of taxes and price controls on essential medicines and weak or absent intellectual property laws and regulatory frameworks. Enabling public policies that create adequate health sector infrastructure, including medical facilities, diagnostic systems and medical service delivery systems, is also key to attracting private sector investment.^43^

As African pharmaceutical manufacturers increasingly develop their own products, there is also an increased need for local regulatory expertise, and this needs to be developed to attract businesses.^3^ New institutions can develop with guidance from established regulatory bodies, such as the Food and Drugs Board in Ghana.^44^

External and international funding remains critical to the sustainability of research and innovation systems in many African countries. However, there are cases where sub-Saharan African health ministries have been overwhelmed with donor requests to fund research activities, while neighbouring countries, with equally substantive disease burdens, have a paucity of funding.^45^

### Initiatives and coalitions

This paper highlights a selection of the many commendable initiatives that target improved research capacity in Africa. Effective stimulation of investment, in such activities, is critical to address many of the issues outlined above and to improve research capacity.

Traditional incentives for investment in product development often rely on having an environment with a well-established infrastructure. For countries with no significant research capacity, the priority needs to be on developing infrastructure and education, oriented towards long-term achievements, to train the researchers of tomorrow. For countries with some research capacity already in place, investment should focus on further developing centres of excellence, as is the focus of the World Bank African Higher Education Centers of Excellence project.

International partners can also risk engaging in a manner that hinders capacity development, despite best intentions. This is a problem for local and international donors, academic institutions and non-governmental organisations. Partnerships can be imbalanced and inequitable, favouring the careers and priorities of researchers based outside of Africa.^46^ Power imbalances require specific interventions to address them, such as having core principles to guide research partnerships. These include the work of the Canadian Coalition for Global Health Research, Commission for Research Partnerships with Developing Countries (KFPE) and the Research Fairness Initiative.^47^

African-led solutions can address such imbalances and weaknesses in infrastructure. The ANDI was established in 2008 as a pan-African agency with a mission to promote and sustain African-led health innovation to address the health needs of the poor, and has contributed significantly to the innovation space in Africa.^34 48^ In 2015, the Alliance for Accelerating Excellence in Africa (AESA) was created by the African Academy of Sciences (AAS) and the New Partnership for Africa’s Development (NEPAD) Agency, with the support of DFID, the Wellcome Trust and the Bill & Melinda Gates Foundation. The AESA is a vehicle to manage research funding and provide research leadership for the continent of Africa, supporting the training of scientists and driving Africa’s research agenda. The ‘Coalition for Research and Innovation’ (CARI) is another platform within Africa from which a coalition of African leaders, African philanthropists and international funders can build a highly coordinated, well-funded and innovative African R&D community. The Biosciences Eastern and Central Africa is a further NEPAD-African Union initiative convening scientists from the two regions to work on common challenges in food and nutritional security.^49^ The AAS 2018 report on ‘Africa Beyond 2030’^49^ outlines the current landscape of such initiatives across the region.^49^

**Figure 2:**
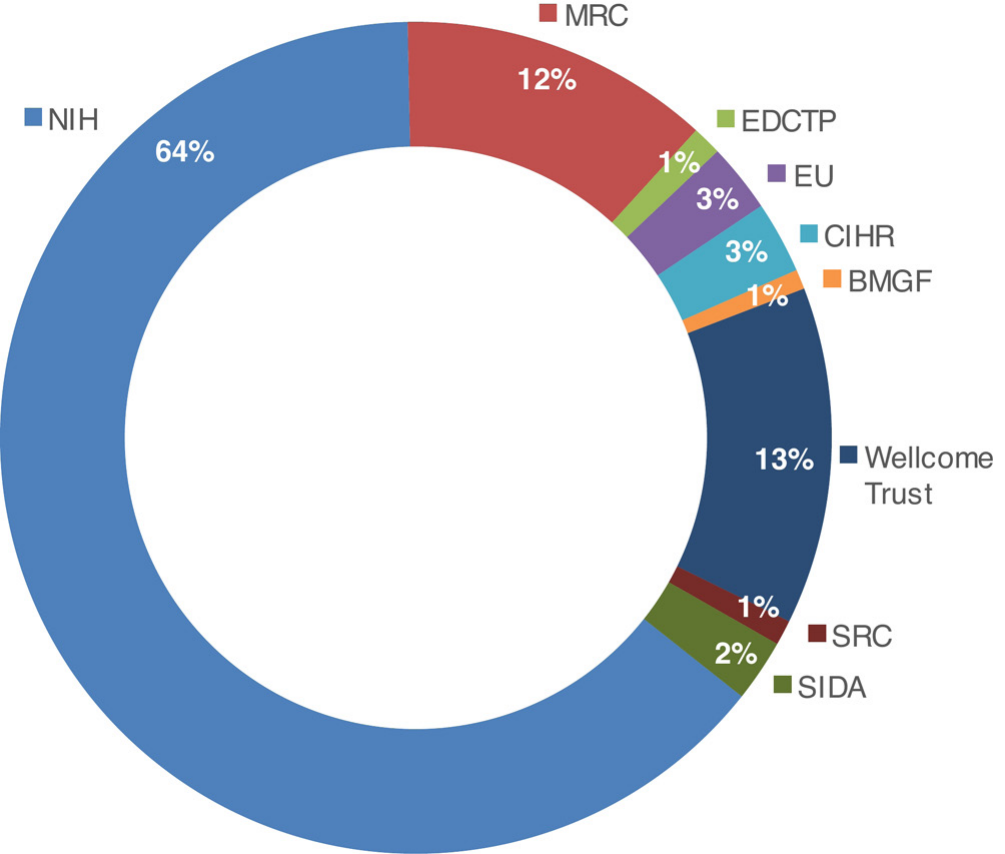
Research programmes funded by organisations in Africa in 2015. Source.^24^ Organisations listed in figure: BMGF, Bill & Melinda Gates Foundation; CIHR, Canadian Institutes of Health Research; EDCTP, European and Developing Countries Clinical Trials Partnership; EU, European Union; MRC, UK Medical Research Council; NIH, US National Institutes of Health; SIDA, Swedish International Development Cooperation Agency; SRC, Swedish Research Council; Wellcome Trust.

Such initiatives extend to suggestions for coordinating pooled funding initiatives for African R&D. These include a pan-African fund with contributions from governments, donors and the private sector.^5^ Such mechanisms need ethical governance frameworks representative of local priorities and that prevent countries receiving disproportionate amounts of support compared with their neighbours. A better understanding of how existing investments work can also support more effective funding. World RePORT is one way to do so, as an online database that maps research projects funded around the world. World RePORT provides information on investments and partnerships from some of the largest biomedical research funders.^24^ Figure 2 illustrates the percentage of research programmes within Africa funded by the global research funders who provided data.

## Conclusion

Science, technology and innovation are key to Africa’s future. Most experience on life science research policy development, however, comes from high-income countries in Europe and North America where many elements of infrastructure are already in place.^50^ Clear development strategies and innovative financing mechanisms could encourage public, private and international investment. For this to be sustainable, incentives need to be relevant to the settings where they are used.^51^

Many initiatives are already in place in Africa, and momentum should build behind these to support R&D capacity building. Doing so requires a collaborative approach that ensures African leadership and ownership. Initiatives such as CARI and AESA are Africa-led, Africa-centred and Africa-specific platforms for collaboration. With domestic and international support, these and other initiatives can address development challenges in Africa and foster long-term sustainable development of excellence and leadership in science, research and innovation.
