# Supplementary material for: Investing in health R&D: where we are, what limits us, and how to make progress in Africa
Source: BMJ Glob Health. 2019 Mar 4;4(2):e001047. doi: 10.1136/bmjgh-2018-001047 (PMC6407556; doi:10.1136/bmjgh-2018-001047)
Supplement: Supplementary data [file bmjgh-2018-001047supp003.pdf]

Appendix A – Methods

This analysis divided its methodological approach into two phases: a semi-systematic literature review and interviews.

The first phase involved collecting information and evidence on health science research output, research capacity building and investment in health science research in Africa. This was undertaken through a semi-systematic literature review.[9] We reviewed relevant peer-reviewed articles with the use of MEDLINE (PubMed), Embase (Ovid) and Web of Science. Search terms included 'health science research', 'research output', 'research capacity building', 'investment'. Literature published up to May 2017 was examined and restricted to papers published in English. Additional, non-peer reviewed, 'grey' literature was included following identification through the Google search engine, and from citations in several key papers and publication archives on relevant websites.

The second phase of this review used a sample of informant interviews with experts in their fields. Interviews were conducted with nine experts, chosen using a judgement sampling approach, via telephone or email correspondence to learn more about particular areas of interest.[10] Experts were selected from governmental organisations such as the National Institutes of Health (US) and the Department for International Development (UK), non-governmental organisations such as the Wellcome Trust, Bill and Melinda Gates Foundation, the World Economic Forum, and academia including the Alliance for Accelerating Excellence in Science in Africa and the Liverpool School of Tropical Medicine. The primary objectives of these interviews were to examine the key gaps and challenges in the current research and

capacity building environment. Further objectives were to: understand the level, adequacy and sustainability of funding for research capacity building, the types of monitoring and evaluation mechanisms in place to assess programme efficacy and the possible funding mechanisms to encourage sustainability.

These interviews enriched the scope of this review. The discussion in this article are based on the authors' assessment, and do not necessarily reflect the opinions of the experts consulted in the process. Interview questions included:

1. What in your view are the key gaps in the current capacity building environment?
2. What is the current level, adequacy and sustainability of the funding for research capacity building?
3. In your view what are the main obstacles to building local infrastructure and research capacity?
4. Monitoring and evaluation mechanisms in place to assess programme efficacy?
5. Do you know of any literature looking at economic returns on investment within Africa?
6. Possible funding mechanisms to encourage sustainability? Fund – where all contribute but what are the incentives?
7. Examples of countries who have used investments to develop good research capacity and output.
