# Supplementary material for: Investing in health R&D: where we are, what limits us, and how to make progress in Africa
Source: BMJ Glob Health. 2019 Mar 4;4(2):e001047. doi: 10.1136/bmjgh-2018-001047 (PMC6407556; doi:10.1136/bmjgh-2018-001047)
Supplement: Supplementary data [file bmjgh-2018-001047supp001.pdf]

Additional File A

File format – Word document .docx

Title: GDP per capita and GERD as % of GDP by African country in 2014 (or latest available year)

Description: Table detailing the GDP per capita, GERD as % of GDP and GERD per country in 2014 or latest available year.

Source: UNESCO Institute for Statistics [http://uis.unesco.org]

Country	GDP per capita - PPP$	GERD as % of GDP	GERD (PPP) millions
Algeria*	14,687	0.07	242
Egypt§§§	10,891	0.68	6,402
Libya	14,154	-	-
Morocco∞∞	7,821	0.71	1,484
Tunisia§§§	11,397	0.64	806
Angola∞∞∞	7,371	-	-
Benin	2,110	-	-
Botswana§	15,807	0.25	77
Burkina Faso∞	1,659	0.2	40
Burundi∞∞∞∞	736	0.12	8
Cabo Verde∞∞∞∞	6,690	0.07	2
Cameroon	3,123	-	-
Central African Republic	597	-	-
Chad	2,171	-	-
Comoros	1,435	-	-
Congo	6,368	-	-
Cote d'Ivoire*	3,496	-	-
DRC∞	783	0.08	28
Djibouti	3,282	-	-
Equatorial Guinea	30,041	-	-
Eritrea	1,411	-	-
Ethiopia§§	1,626	0.6	786
Gabon∞	20,010	0.57	128
Gambia∞∞∞∞	1,636	0.13	4
Guinea	1,207	-	-
Guinea-Bissau	1,453	-	-
Kenya∞∞	3,083	0.79	788
Lesotho∞∞∞∞	2,648	0.01	1
Liberia	836	-	-
Madagascar§§§	1,459	0.01	5
Malawi∞∞	1,183	1.06	-
Mali∞∞	2,428	0.67	153
Mauritania	3,886	-	-
Mauritius§	19,480	0.18	39

Mozambique∞∞	1,186	0.42	92
Namibia∞∞	10,414	0.14	26
Niger*	954	-	-
Nigeria**	5,992	0.22	1,375
Rwanda∞	1,759	-	-
Sao Tome and Principe	3,188	-	-
Senegal∞∞	2,431	0.54	150
Seychelles*	27,273	0.3	4
Sierra Leone	1,591	-	-
Somalia	-	-	-
South Africa§	13,165	0.73	4,824
South Sudan	1,850	-	-
Sudan*	4,173	0.3	281
Swaziland	8,427	-	-
Togo§§§	1,460	0.27	27
Uganda∞∞	1,825	0.48	240
Tanzania§§	2,667	0.53	623
Zambia***	3,853	0.28	101
Zimbabwe§	1,794	-	-

2005*, 2007**, 2008***, 2009∞, 2010∞∞, 2011∞∞∞, 2012§, 2013§§, 2014§§§
