# Supplementary material for: Investing in health R&D: where we are, what limits us, and how to make progress in Africa
Source: BMJ Glob Health. 2019 Mar 4;4(2):e001047. doi: 10.1136/bmjgh-2018-001047 (PMC6407556; doi:10.1136/bmjgh-2018-001047)
Supplement: Supplementary data [file bmjgh-2018-001047supp002.pdf]

Additional File B
File format – Word document.docx
Title: Research collaboration in African countries
Description: The level of research collaboration by country detailed by percentage of total research collaboration that takes place within the country, with countries outside Africa and with other countries within the African continent.
Source: Pouris A, Yuh-Shan, Ho.: Research emphasis and collaboration in Africa. *Scientometrics* 2014, 98:2169-2184.

Country	Single country (% of total)	Collaboration outside Africa (% of total)	Collaboration inside Africa (% of total)
South Africa	47	49	3.9
Egypt	57	42	0.63
Tunisia	50	48	1.3
Nigeria	71	23	5.7
Algeria	42	58	1.3
Morocco	40	58	1.8
Kenya	16	78	5.9
Cameroon	21	70	9.3
Uganda	15	79	5.7
Tanzania	14	81	5.1
Ethiopia	29	66	5
Ghana	26	70	4.8
Senegal	16	76	8
Sudan	31	65	4
Malawi	14	76	10
Burkina Faso	8.5	83	8.5
Zimbabwe	16	66	18
Cote d'Ivoire	29	68	3.7
Benin	13	78	10

Madagascar	8.7	90	1.4
Zambia	5.5	89	5.4
Botswana	26	54	19
Libya	29	61	10
Mali	5.9	89	5
Mozambique	4.3	89	6.9
Gabon	3.7	92	4.2
Congo	7.6	84	8.6
Gambia	5.5	93	1.6
Niger	7.5	76	16
Namibia	11	75	14
DRC	6.5	89	4.5
Rwanda	5.5	85	9.1
Mauritius	36	60	4
Togo	23	67	10
Swaziland	19	52	29
Angola	3.4	91	5.2
Seychelles	3.6	95	1.8
Guinea Bissau	1.8	98	0
Guinea	3.7	91	5.5
CAD	6.7	83	10
Mauritania	4.7	66	29
Eritrea	9.3	90	1.2
Lesotho	8.4	54	37
Sierra Leone	10	80	10
Chad	6.8	82	11

Burundi	1.5	96	3
Cape Verde	0	100	0
Djibouti	12	85	3.8
Liberia	0	96	4.3
Comoros	0	80	20
Equatorial Guinea	0	100	0
Somalia	0	75	25
Sao Tome and Principe	0	100	0
